# Microhardness and elemental analysis of ion-releasing restoration/ dentin interface following enzymatic chemomechanical caries excavation

**DOI:** 10.1186/s12903-024-04304-8

**Published:** 2024-05-19

**Authors:** Rana E. Al-Wakeel, Hamdi H. Hamama, Dina S. Farahat, SA El-Negoly

**Affiliations:** 1 Department of Dental Biomaterials, Faculty of Dentistry, Mansoura University, Mansoura City, Egypt; 2 Department of Conservative Dentistry, Faculty of Dentistry, Mansoura University, Mansoura City, Egypt; 3 Faculty of Dentistry, New-Mansoura University, New-Mansoura City, Egypt

**Keywords:** Glass ionomer, Bioactive, Glass hybrid, Chemomechanical, Remineralization

## Abstract

**Background:**

This study was conducted to compare chemical, elemental and surface properties of sound and carious dentin after application of two restorative materials resin-modified glassionomer claimed to be bioactive and glass hybrid restorative material after enzymatic chemomechanical caries removal (CMCR) agent.

**Methods:**

Forty carious and twenty non-carious human permanent molars were used. Molars were randomly distributed into three main groups: Group 1 (negative control) - sound molars, Group 2 (positive control) - molars were left without caries removal and Group 3 (Test Group) caries excavated with enzymatic based CMCR agent. After caries excavation and restoration application, all specimens were prepared Vickers microhardness test (VHN), for elemental analysis using Energy Dispersive Xray (EDX) mapping and finally chemical analysis using Micro-Raman microscopy.

**Results:**

Vickers microhardness values of dentin with the claimed bioactive GIC specimens was statistically higher than with glass hybrid GIC specimens. EDX analysis at the junction estimated: Calcium and Phosphorus of the glass hybrid GIC showed insignificantly higher mean valued than that of the bioactive GIC. Silica and Aluminum mean values at the junction were significantly higher with bioactive GIC specimens than glass hybrid GIC specimen. Micro-raman spectroscopy revealed that bioactive GIC specimens showed higher frequencies of v _1_ PO _4,_ which indicated high level of remineralization.

**Conclusions:**

It was concluded that ion-releasing bioactive resin-based restorative material had increased the microhardness and remineralization rate of carries affected and sound dentin. In addition, enzymatic caries excavation with papain-based CMCR agent has no adverse effect on dentin substrate.

**Supplementary Information:**

The online version contains supplementary material available at 10.1186/s12903-024-04304-8.

## Introduction

Developing an alternative for dental amalgam has been an essential desire in the last decades. Failure of existing composite resin restorations mainly occurs due to polymerization shrinkage, marginal leakage and secondary caries. Modern composite restoration can achieve acceptable clinical success through isolation of the tooth from saliva and moisture, using bonding agents and building the restoration incrementally in order to reduce polymerization shrinkage. Unfortunately, these procedures are time-consuming and technique sensitive for both dentist and patient. Since the mid-1980s, the increase in demand for tooth-colored restorations has led to dental composites becoming the most widely used restorative material world-wide. Several efforts have continuously been made to refine the materials. One of these attempts is the creation of dental adhesives that adheres to tooth structures, with the goal of eliminating microleakage and its consequences at the restoration/tooth structure interface, as well as retaining restorations and preserving tooth structure [1]. Nevertheless, bonding of resin composite to tooth substrate is dependent on time consuming ‘technique-sensitive’ micromechanical adhesive mechanism, innovations were created by combining the adhesive system and resin composite in a single step technique [2]. 

In order to overcome the drawbacks of direct posterior composite restorations, an altered ion releasing self-adhesive restorative material with claimed bioactivity was introduced [3]. The manufacturer claims that it is bioactive ionic resin matrix that releases and recharges a sufficient amount of calcium, phosphate, and fluoride ions and reacts to the continuous pH changes in the mouth [4]. While the approval document obtained from the US Food and Drug Administration (510 k123265) described this material as a self-adhesive dual cured resin modified glass ionomer (RMGI), it is claimed to combine the water friendly properties, more release and recharge of calcium, phosphate and fluoride ions than glass ionomers with the durability and improved physical properties of resin- based materials.

In the past decades, dental caries prevention and treatment have changed. Rotary instrumentation in caries removal has a main drawback, through the vibration caused by bur rotation frequency which is uncomfortable for patients. In the last decades, Minimally Invasive Caries Removal Technique (MICRT) has been one of the most important applications of minimal intervention dentistry concepts [5]. Laser ablation, air abrasion, sono-abrasion and chemomechanical agents were incorporated recently in the removal of infected dental tissues and provided significant advancements in MICRTs. Selective removal of caries-infected tissue and leaving the caries-affected tissues intact, is the main aim of MICRTs. Carries affected dentin is characterized by no destruction in the collagen matrix, no bacterial penetration and demineralization of the intertubular dentin. On the contrary, in caries infected dentin denaturation of collagen matrix, bacterial penetration and distortion of the dentinal tubules microstructure occurs [6]. 

Chemomechanical caries removal (CMCR) is considered one of the most conservative means of caries excavation using minimal invasive caries removal techniques. The concept of removing caries using a chemical solution was first introduced by Goldman in 1976. These techniques are mainly dependent on the selective removal of caries-infected dentin, while leaving the caries-affected tissue intact. CMCR technique’s main principle is to alter the chemical composition of the carious tooth, which leads to its softening, and then removed mechanically using a hand instrument. These techniques are very important to provide more infection control precautions, as they don’t generate any aerosols or droplets to be suspended in the air, which mainly led to several viral infections; especially our recent pandemic Coronavirus COVID-19, primarily spreads through the respiratory tract, by droplets, respiratory secretions and direct contact [7]. CMCR agents can be divided into two main groups: sodium hypochlorite (NaOCl) based agents and the enzymatic-based agents which are Papain-enzymatic based CMCR agent and the experimental material, ‘Biosolv’ [8]. 

Different studies have been conducted before to clarify the effect of these agents on the caries-affected dentin. The majority of these studies have been conducted on sodium hypochlorite (NaOCl) based agents; however, very few studies have been conducted on the enzymatic-based chemomechanical caries removal agents [9]. This study was conducted to evaluate the effect of the bioactive restorative material on caries affected dentin after removing the caries using Papain-enzymatic based CMCR agent. In light of the available literature, there is scared information about remineralizing effect of bioactive restorations on caries-affected dentin following enzymatic caries excavation method. Consequently, the rationale behind conducting this study was to evaluate the remineralizing power of two ion-releasing restorative materials (GIC- and Resin-based) on caries-affected dentin after enzymatic CMCR method.

The present study was conducted to compare between an ion-releasing bioactive resin-based restorative material applied to sound and caries affected dentin (previously removed by using CMCR technique) and a glass hybrid restorative material (Glass-hybrid GIC Fil). Vickers microhardness changes in dentin after CMCR was measured. Elemental analysis of hybrid-like layer was done using an energy dispersive x-ray (EDX) mapping. Chemical analysis of dentin was done using a micro-raman microscopy. The null-hypotheses tested for this study were that there were no differences: in microhardness and elemental analysis of dentin using two different restorative materials under various dentin conditions.

## Materials and methods

### Materials

In this study, two restorative materials were investigated as follows; ion-releasing bioactive resin-based restorative material (Activa, Pulpdent, and Watertown, MA, USA) and a glass hybrid restorative material (Equia-Forte Fil, GC Corp, Tokyo, Japan). Also, an enzymatic based CMCR agent was used. Each restorative material was used according to manufacturer’s instructions. The full description of the materials used is presented in Table 1.

### Study design

**Fig. 1:**
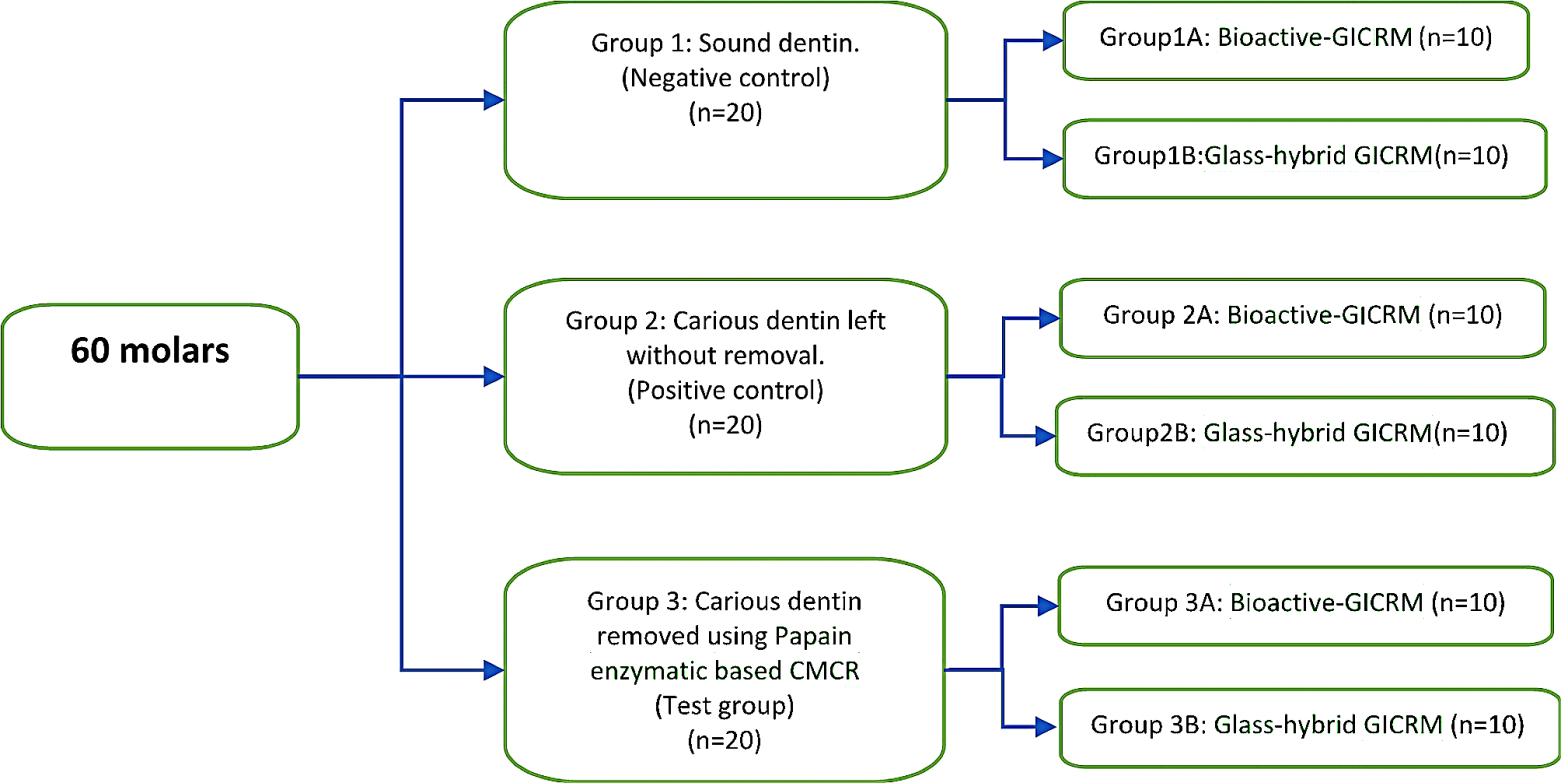
Schematic diagram showing the treatment groups

The sample size was estimated using the G power 3.1.9.2 calculator program from the University of Kiel, Germany (http://www.gpower.hhu.de/). Sample size was estimated on mean of previously published study by Hamdi et al. [10] Sixty extracted human permanent molars (forty carious and twenty non-carious) were used. All the study protocol steps were approved by The Ethical committee of Faculty of Dentistry, Mansoura University with reference number (M03060421). The molars were randomly distributed into 3 groups Group 1 (negative control) - sound molars, Group 2 (positive control) - molars were left without caries removal and Group 3 (Test Group) caries excavated with enzymatic based CMCR agent (Papacarie). Each group was subdivided into 2 subgroups relative to the type of restorative material used; an ion-releasing bioactive resin-based and a glass hybrid restorative materials as shown in Fig. 1.

**Table 1:** Describes the materials used in the study

Material	Composition	Manufacturer
Activa Bioactive GIC Restorative	Powder: silanated bioactive glass and calcium, silanated silica, and sodium fluoride Liquid: diurethane modified by the insertion of a hydrogenated polybutadiene and other methacrylate monomers, modified polyacrylic acid, and water Filler: 56 wt% (50% bioactive glass and ca.,7% silica)	Pulpdent (Watertown, MA, USA)
Equia Forte restorative material (Glass-hybrid GIC fil)	Powder: 95% strontium fluoro-alumino-silicate glass, including the newly added highly reactive small particles, and 5% polyacrylic acid powder. Liquid: 40% polyacrylic acid, 50% distilled water and 10% polybasic carboxylic acid.	GC Corp, (Tokyo, Japan)
Equia Forte coat	50% Methyl methacrylate, colloidal silica, 0.09% camphorquinone	
Cavity Conditioner	77% distilled water, 20% polyacrylic acid, 3% aluminumchloride hydrate .	
Fuji Varnish (Resin Protective coating)	Isopropyl acetate, acetone, cornmint oil and cinnamaldehyde.	
Enzyme-based CMCR agent; Papacarie	Papain, chloramine and tioluidine blue	Formula & Acao (Brazil)

The occlusal enamel was removed to expose mid-coronal dentin. This was performed by cutting perpendicular to the long axis of the tooth using a low-speed diamond disc (Isomet, Buchler, USA) under water irrigation. The prepared dentin surface was examined using a stereomicroscope (Olympus model SZ-PT, Tokyo, Japan) at x40 magnification to detect any remaining enamel. The teeth were hand polished using #600 silicon carbide sandpapers under running water to expose a flat dentin surface with standardized smear layer. Each tooth was embedded in self-cured acrylic resin (Acrostone, Anglo-Egyption Company, Cairo, Egypt) in plastic cylinders of 10-mm height and 15-mm diameter with the flat dentin surface exposed. The exposed dentin surface was washed with deionized water, dried with compressed air, conditioned by dentin conditioner (Cavity Conditioner, GC, Corp, Tokyo, Japan) for 20 s according to the manufacturer instructions, then rinsed with water, and dried by cotton pellet to avoid desiccation of the dentin surface.

### Chemomechanical caries excavation of carious dentin

Papain-enzymatic based CMCR gel was applied to the decayed tissue (ICDAS 5) and left for 30 to 60 s. When the gel appeared cloudy not shiny (due to presence of decayed tissue), softened carious dentin was removed using the blunt back of a non-sharp excavator. This process was repeated till observation of the unique CMCR end-excavation point which is: failure of the gel to gain turbidity. Afterwards, rinsing of the surface and gentle drying was performed. The surface was left shiny, hard and decontaminated before proceeding to the restoration of the cavity.

### Specimen preparation

The restorative material was applied to the prepared dentin in a cylindrical mold of 5-mm height and 6-mm diameter. The materials were inserted according to the manufacturer’s instructions. The encapsulated Glass-hybrid GIC material was prepared by mixing of the capsule for 10 s in the amalgamator (CM-II, GC, Japan), the mixed cement was applied to the mold using the cement applier. After 20 min, the specimen was removed from the mold, then coated with the glass hybrid coat and light cured for 20 s. Bioactive-GIC restorative material was applied to dentin surface (according to the manufacturer’s instructions) in 2 mm increments, light cured for 20 s and specimens were coated from all sides by a layer of varnish (GC Fuji Varnish). All specimens were covered separately using damped cotton with distilled water and stored for 24 h at 37 °C, to prevent dehydration and simulate the oral condition.

### Microhardness changes of dentin after chemomechanical caries removal

**Fig. 2:**
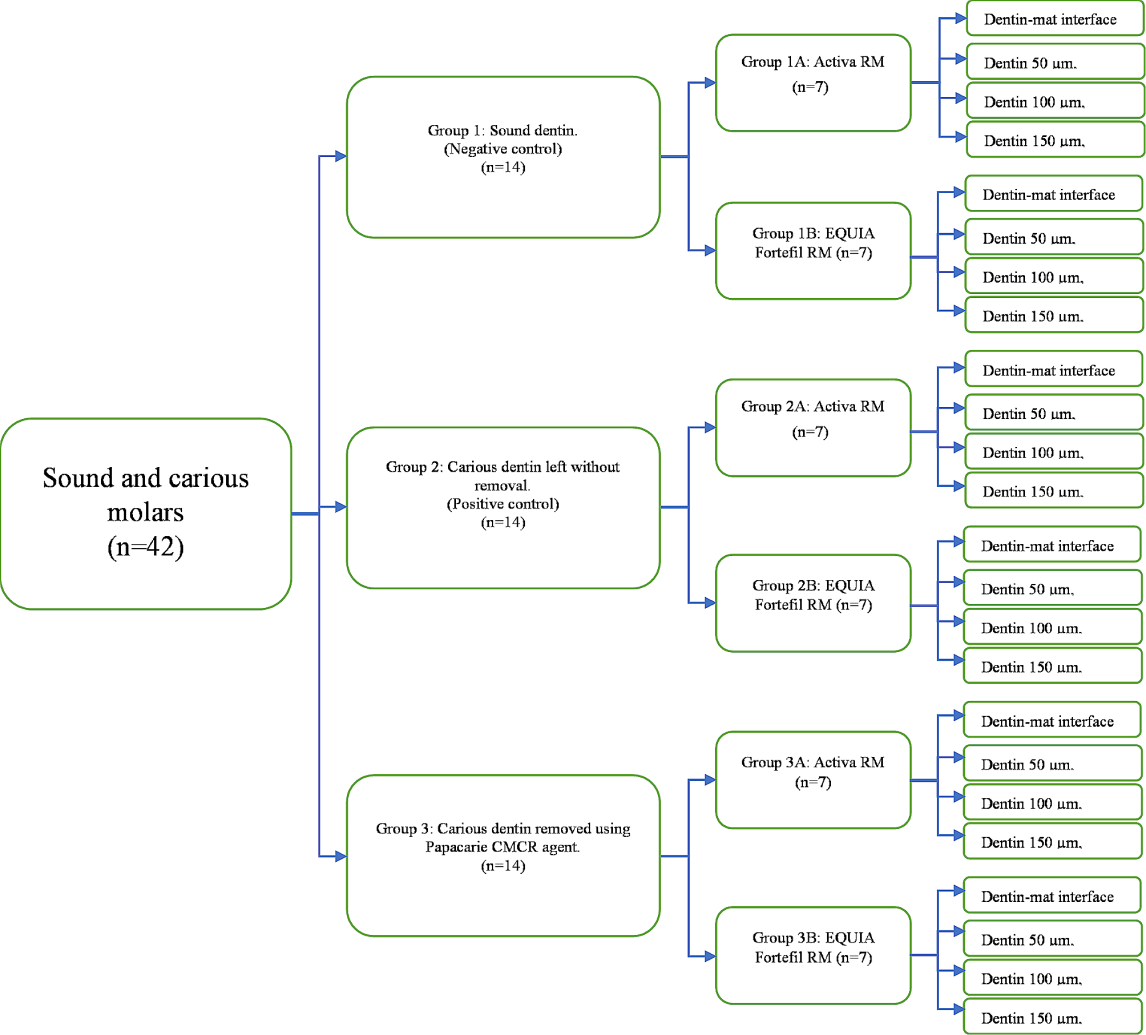
Schematic diagram showing the treatment groups regarding microhardness examination

Fourty two specimens from the previously restored molars (28 carious and 14 non-carious specimen) as shown in Fig. 2. All specimens were prepared for Vickers hardness testing, by sectioning the specimens into two halves longitudinally in the mesiodistal plane using an automated diamond saw (Isomet, Buchler, USA) under copious water coolant to gain two halves. Afterwards, specimens were hand polished using #600 silicon carbide sandpapers under running water and ultrasonically cleaned. Custom made cylindrical plastic molds were prepared using plastic syringes and filled with self-cured acrylic resin (Acrostone, Anglo-Egyption Company, Cairo, Egypt). Each half of the specimens was embedded in the resin with restoration and dentin facing upward, exposed and parallel to the horizontal plane of the acrylic resin. Specimens were stored by application of a damp fibreless laboratory napkin on top of the tested surface and removed immediately before testing, to avoid their dehydration.

Vickers micro-hardness was measured at the restoration-tooth interface and at three levels in the dentin (50 μm, 100 μm and 150 μm) using a Vickers hardness tester (HV-1000 LTD, Jinan Precision Testing Equipment Co. Ltd., China). Pyramidal diamond indenter was loaded to 50 g (0.49 N) for a dwell time of 10 s leaving an indentation on the polished surface. Each indentation was separated from the following one by at least 50 μm. The two diagonals of each indentation were measured, and the Vickers hardness number was automatically calculated.

### Elemental analysis of restoration/tooth interface using EDX

**Fig. 3:**
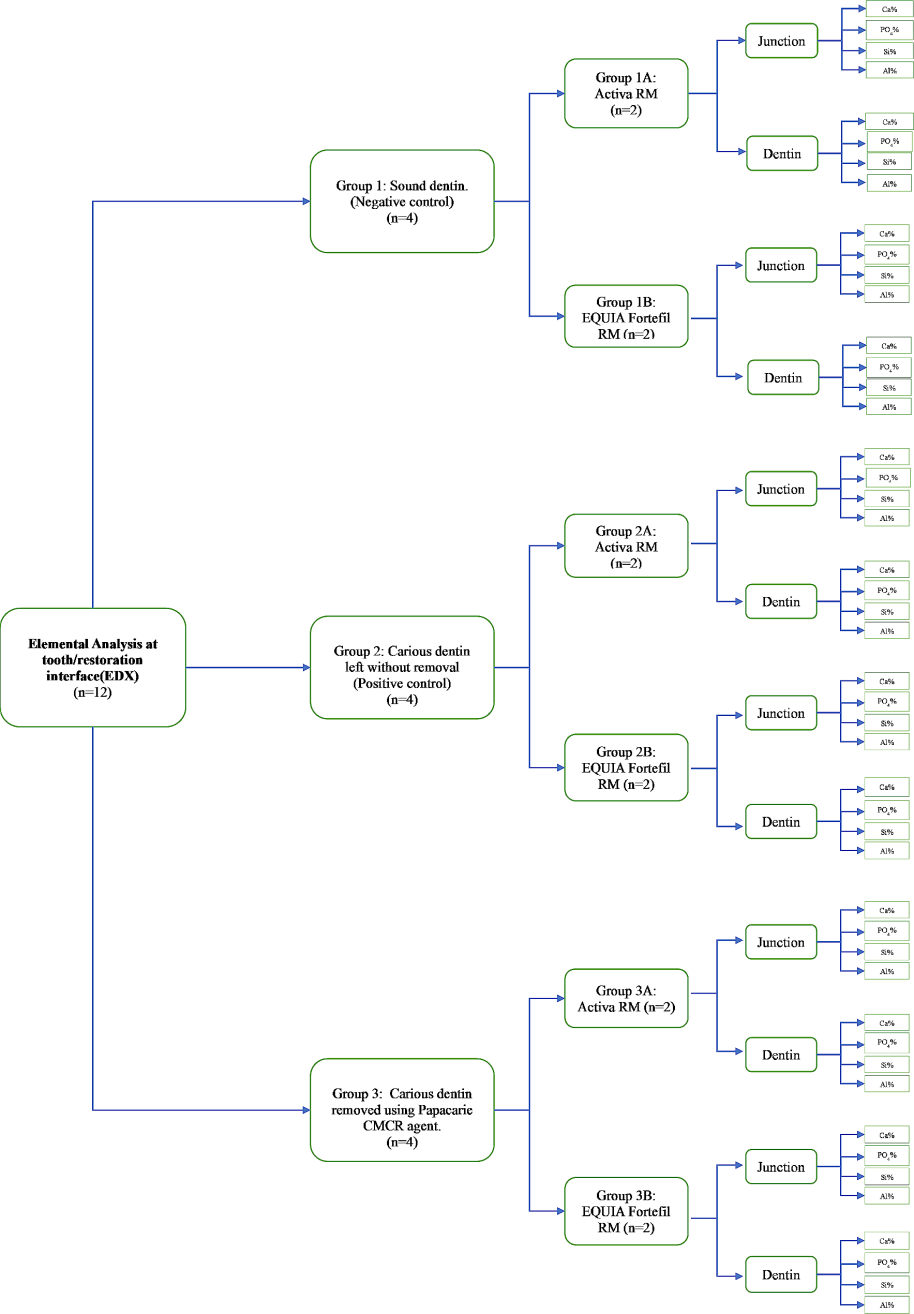
Schematic diagram showing the treatment groups regarding EDX

Twelve specimens were used for elemental analysis (*n* = 2 for each subgroup). Each specimen was prepared as previously prepared in Vicker’s microhardness testing. Each half of the specimen was examined separately (Fig. 3).

Specimens were subjected to elemental analysis under conditions of low vacuum for acceleration voltage 20.0 ∼ 30.0 kv using large field detector with working distance 15 ∼ 17 mm, calcium, silica, phosphorous and aluminum weight% were detected at tooth/restoration interface and dentin using ‘area selection’ function of EDX Oxford software attached to a scanning electron microscopy.

### Chemical analysis of dentin using micro-raman microscopy

Specimens were prepared as previously in the last two tests (one specimen for each subgroup (*n* = 6) were analyzed using micro-Raman spectroscopy (RAMANtouch, Nanophoton Co., Ltd., Osaka, Japan), to detect the mineral composition at the restorative/tooth interface point. The excitation light used was a green laser beam with a wavelength of 532 nm in the spectral range of 400 to 1200 cm^− 1^. Micro-Raman spectra were obtained using a×50 objective to focus the laser beam on the detected location. Origin version 6.1 (OriginLap, Northampton, MA, USA) and Peakft version 4.0 (Aspire Sofware International, Ashburn, VA, USA) were used to analyze Raman spectra. The ratio of v_1_ PO4 ^3−^ (960 cm^− 1^), v_2_ PO4 ^3−^ (431 cm^− 1^), and v_4_ PO4 ^3−^ (589 cm^− 1^) and B-type carbonate v_1_ CO3 ^2−^ (1072 cm^− 1^) was obtained, but v_1_ PO4 ^3−^ (960 cm^− 1^) and B-type carbonate v_1_CO3 ^2−^ (1072 cm^− 1^) were generalized to analyze the change in mineral composition among the specimens.

### Statistical analysis

The sample size was estimated using the G power 3.1.9.2 calculator program from the University of Kiel, Germany (http://www.gpower.hhu.de/). The data obtained from the experiments was collected and tabulated. All the data were subjected to testing of normal distribution and appropriate statistical analysis was conducted.

## Results

### Microhardness test

The mean and standard deviation (SD) Vickers hardness values of sound dentin (negative control group), carious dentin (positive control group) and residual dentin following caries removal via papain-enzymatic based CMCR agent are shown in Table 2. The outcome of the Kolmogorov–Smirnov normality test showed that data followed the normal distribution and accordingly parametric statistical analysis was performed. The outcome of three-way ANOVA revealed that the variables; ‘teeth condition’, ‘material used’ and ‘site’ significantly affect the Vickers micro-hardness of the dentin (*p* < 0.05). Moreover, the interaction between the three variables exhibited significant effect on Vickers micro-hardness (*p* < 0.05).

Vickers hardness of Sound dentin -Bioactive-GIC group at junction site showed the highest value and was statistically significant different from all the test groups (*p* < 0.05). Vickers hardness of the Carious dentin-Glass-hybrid GIC group at 50 μm dentin showed the lowest value and had significant difference with all of the test groups except Carious dentin-Glass-hybrid GIC group at junction site and at dentin 100 μm, CMCR-Bioactive-GIC group at 100 and 150 μm dentin.

In Sound -Glass-hybrid GIC group Vickers hardness at junction was significantly higher than the three other groups (*p* < 0.05). While Vickers hardness values at dentin 50 μm, 100 μm and 150 μm showed insignificant difference between each other (*p* > 0.05). Similarly, Vickers hardness in Sound - Bioactive-GIC group at junction showed significant different with the three other groups, Vickers hardness at 50 μm, 100 μm and 150 μm dentin showed insignificant difference between each other (*p* > 0.05). Comparing the two sound groups showed insignificant differences between all groups (*p* > 0.05).

Vickers microhardness results of Caries- Glass-hybrid GIC group showed that, there is a significant difference between Caries- Glass-hybrid GIC group at 50 μm dentin and 150 μm dentin subgroup (*p* < 0.05). In addition, statistically insignificant difference was found between Caries -Glass-hybrid GIC group at junction and Caries -Glass-hybrid GIC group at 100 μm dentin (*p* > 0.05). No significant difference was found between Caries-Bioactive-GIC groups at junction, 50 μm dentin, 100 μm dentin and 150 μm dentin. Comparing the two caries groups, significant differences were found between Caries- Glass-hybrid GIC group at 50 μm and Caries -Bioactive-GIC groups at junction, 50 μm dentin, 100 μm dentin and 150 μm dentin (*p* < 0.05).

**Table 2:** Microhardness of sound and residual dentin after caries removal

Substrate (*n* = 56)	Material (*n* = 84)	Site (*n* = 42)	Mean ± SD
Sound	Glass-hybrid GIC	Junction	78.97 ± 10.46^b, c^
		Dentin(50 μm)	58.69 ± 12.60^d, e, f, g^
		Dentin(100 μm)	61.51 ± 7.96^f, e, d^
		Dentin(150 μm)	60.26 ± 9.52^f, e, d^
	Bioactive-GIC	Junction	106.19 ± 13.17^a^
		Dentin(50 μm)	69.36 ± 4.33^b, c, d^
		Dentin(100 μm)	65.86 ± 3.24^c, d. e^
		Dentin(150 μm)	64.09 ± 2.80^c, d, e, f^
Carious	Glass-hybrid GIC	Junction	48.40 ± 4.78^h, g, f^
		Dentin(50 μm)	35.17 ± 5.2^h^
		Dentin(100 μm)	43.14 ± 3.42^h, g^
		Dentin(150 μm)	52.37 ± 6.67^g, f, e^
	Bioactive-GIC	Junction	53.63 ± 9.02^g, f, e, d^
		Dentin(50 μm)	56.38 ± 7.25^g, f, e, d^
		Dentin(100 μm)	58.68 ± 6.88^g, f, e, d^
		Dentin(150 μm)	63.28 ± 4.6^c, d, e, f^
CMCR	Glass-hybrid GIC	Junction	84.39 ± 9.59^b^
		Dentin(50 μm)	59.02 ± 10.10^g, f, e, d^
		Dentin(100 μm)	67.03 ± 11.75^c, d, e^
		Dentin(150 μm)	63.79 ± 12.00^c, d, e, f^
	Bioactive-GIC	Junction	52.80 ± 9.09 ^g, f, e^
		Dentin(50 μm)	54.94 ± 7.56 ^g, f, e, d^
		Dentin(100 μm)	49.40 ± 6.57^h, g, f^
		Dentin(150 μm)	49.41 ± 4.41^h, g, f^

Groups identified by different superscripts were significantly different at *p* < 0.05, *n* = 30.

CMCR- Glass-hybrid GIC group at junction showed significant difference with all the CMCR sub-groups. While there were insignificant differences between the remaining groups. CMCR-Glass-hybrid GIC group at 100 μm dentin showed significant difference with CMCR-Bioactive-GIC group at 100 μm dentin (*p* < 0.05). CMCR-Glass-hybrid GIC group at 150 μm dentin showed significant difference with CMCR-Bioactive-GIC group at 150 μm dentin (*p* < 0.05).

### Elemental analysis at tooth/restoration interface

**Fig. 4:**
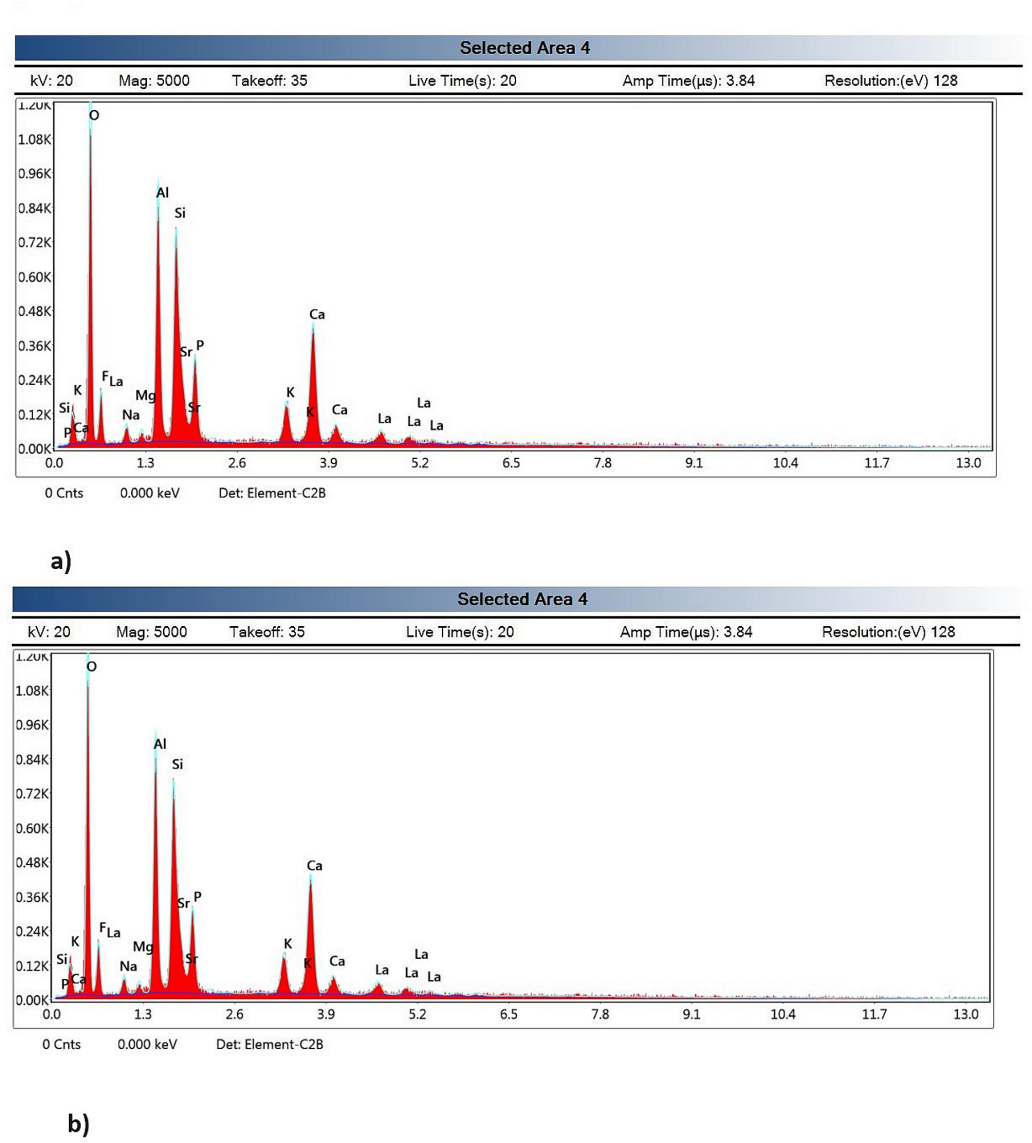
(**a**): Representing the elemental analysis results of the sound- Glass hybrid -junction group. (**b**): Representing the elemental analysis results of the CMCR- Bioactive GIC-junction group

The EDX elemental analysis of Calcium, Silica, Phosphorous and Aluminum is shown in Table 3; Fig. 4(a & b). The results of three-way ANOVA regarding Calcium levels revealed that substrate condition and material used had insignificant effect on Calcium levels (*p* > 0.05). While site factor had significant effect on Calcium level (*p* < 0.05). The interaction between “substrate-material” and “substrate- site” showed insignificant effect on Calcium levels (*p* > 0.05). While the interaction between “material-site” had significant effect on Calcium level (*p* < 0.05). The interaction between the three factors was insignificant (*p* > 0.05).

Sound-Bioactive-GIC-Dentin, Caries-Bioactive-GIC-Dentin and CMCR-Bioactive-GIC-Dentin groups showed the highest Calcium ratio, these groups were significantly different with all remaining groups, except three groups that showed insignificant differences between them which were: Sound-Glass-hybrid GIC-Dentin, Caries-Glass-hybrid GIC-Dentin and CMCR-Glass-hybrid GIC-Dentin groups. While the lowest Calcium ratios were found in Sound-Bioactive-GIC-Junction, CMCR-Glass-hybrid GIC-Junction and CMCR-Bioactive-GIC-Junction groups these groups were significantly different with all test groups except three groups that were insignificantly different with them which are: Sound-Glass-hybrid GIC-Junction, Caries-Glass-hybrid GIC-Junction and Caries-Bioactive-GIC-Junction groups.

Comparing the sound groups: There were significant difference between Sound-Glass-hybrid GIC-Junction and Sound-Glass-hybrid GIC-Dentin groups, and between Sound-Bioactive-GIC-Junction and Sound-Bioactive-GIC-Dentin groups (*p* < 0.05). Insignificant differences were found between Sound-Glass hybrid GIC-Junction and Sound-Bioactive-GIC-Junction, and between Sound-Glass-hybrid GIC-Dentin and Sound-Bioactive-GIC-Dentin groups (*p* > 0.05). In caries groups: significant difference) was found between Caries-Bioactive-GIC-Junction and Caries-Bioactive-GIC-Dentin group (*p* < 0.05). CMCR groups showed: significant.

**Table 3:** Elemental content of carious, sound and residual dentin after caries removal

Substrate (*n* = 20)	Material (*n* = 20)	Site (*n* = 30)	Mean ± SD Calcium	Mean ± SD Silica	Mean ± SD Phosphorous	Mean ± SD Aluminum
Sound	Glass-hybrid GIC	Junction	0.27 ± 0.22 ^c, d, **e**^	0.07 ± 0.01 ^c^	0.08 ± 0.02^a^	0.09 ± 0.01^c^
		Dentin	0.45 ± 0.03 ^**a**, b^	0.00 ± 0.00 ^d^	0.16 ± 0.02^a^	0.01 ± 0.00^a^
	Bioactive-GIC	Junction	0.18 ± 0.05 ^**e**^	0.16 ± 0.01 ^**a**^	0.04 ± 0.01^a^	0.04 ± 0.00^b^
		Dentin	0.49 ± 0.03 ^**a**^	0.01 ± 0.00 ^d^	0.17 ± 0.01^a^	0.02 ± 0.01^a^
Carious	Glass-hybrid GIC	Junction	0.31 ± 0.02 ^b, c, d, **e**^	0.01 ± 0.01 ^d^	0.12 ± 0.00^a^	0.00 ± 0.00^a^
		Dentin	0.38 ± 0.04 ^**a**, b, c, d^	0.01 ± 0.00 ^d^	0.15 ± 0.02^a^	0.01 ± 0.00^a^
	Bioactive-GIC	Junction	0.24 ± 0.04 ^d, **e**^	0.08 ± 0.01 ^c^	0.08 ± 0.01^a^	0.04 ± 0.00^b^
		Dentin	0.47 ± 0.02 ^**a**^	0.01 ± 0.00 ^d^	0.15 ± 0.01^a^	0.02 ± 0.01^a^
CMCR	Glass-hybrid GIC	Junction	0.18 ± 0.04^**e**^	0.08 ± 0.00 ^c^	0.17 ± 0.24^a^	0.09 ± 0.01^c^
		Dentin	0.41 ± 0.02^**a**, b, c^	0.01 ± 0.00 ^d^	0.13 ± 0.00^a^	0.00 ± 0.00^a^
	Bioactive-GIC	Junction	0.21 ± 0.04 ^**e**^	0.12 ± 0.00 ^b^	0.07 ± 0.01^a^	0.02 ± 0.01^a^
		Dentin	0.48 ± 0.02 ^**a**^	0.01 ± 0.00 ^d^	0.17 ± 0.00^a^	0.00 ± 0.00^a^

Groups identified by different superscripts were significantly different at *p* < 0.05, *n* = 30

differences between CMCR-Glass-hybrid GIC-Junction and CMCR-Glass-hybrid GIC-Dentin, and between CMCR-Bioactive-GIC-Junction and CMCR- Bioactive-GIC- dentin group (*p* < 0.05). Insignificant differences were found between CMCR-Glass-hybrid GIC-Junction and CMCR-Bioactive-GIC-Junction, and between CMCR-Glass-hybrid GIC-Dentin and CMCR-Bioactive-GIC-Dentin groups (*p* > 0.05).

The results of three-way ANOVA regarding Silica levels revealed that a significant effect of substrate condition, material used and the site on Silica levels (*p* < 0.05). The interaction between “substrate-material”, “substrate-site” and “material-site” had also a significant effect on Silica levels (*p* < 0.05). The interaction between the three factors was also highly significant (*p* < 0.05).

Sound-Bioactive-GIC-Junction group had the highest ratio of Silica which showed statistically significant differences with all groups (*p* < 0.05). There were significant differences between Sound-Glass-hybrid GIC-Junction and Sound-Glass-hybrid GIC-Dentin groups and between Sound-Bioactive-GIC-Junction and Sound-Bioactive-GIC-Dentin groups (*p* < 0.05). In Caries group there was significant difference between Caries-Bioactive-GIC-Junction and Caries-Bioactive-GIC-Dentin groups while insignificant difference was found between Caries-Glass-hybrid GIC-Junction and Caries-Glass-hybrid GIC-Dentin groups. CMCR group showed: significant difference between CMCR-Glass-hybrid GIC-Junction and CMCR-Glass-hybrid GIC-Dentin, and between CMCR-Bioactive-GIC-Junction and CMCR-Bioactive-GIC-Dentin groups (*p* < 0.05). Significant differences were found between Sound-Glass-hybrid GIC-Junction group and Sound-Bioactive-GIC-Junction group, between Caries-Glass-hybrid GIC junction group and Caries-Bioactive-GIC-junction and between CMCR-Glass-hybrid GIC-junction group and CMCR-Bioactive-GIC-junction group (*p* < 0.05). Insignificant differences were found between CMCR-Glass-hybrid GIC-Junction and CMCR-Bioactive-GIC-Junction and between CMCR-Glass-hybrid GIC-Dentin and CMCR-Bioactive-GIC-Dentin (*p* > 0.05).

The results of three-way ANOVA regarding Phosphorous levels revealed an insignificant effect of substrate condition and material used on Phosphorous levels (*p* > 0.05), while the site “substrate-material”, “substrate-site” had an insignificant effect on Phosphorous levels (*p* > 0.05), while the interaction between “material-site” had a significant effect on Phosphorous levels (*p* < 0.05). The interaction between the three factors wasn’t significant (*p* > 0.05). There were no significant differences between all the groups (*p* > 0.05).

The results of three-way ANOVA regarding Aluminum levels revealed that substrate condition, material and site factor had a significant effect (*p* < 0.05) on Aluminum levels, The interaction between “substrate-material”, “substrate-site” and “material-site” had a significant effect on aluminum levels (*p* < 0.05). The interaction between the three factors was also high significant (*p* < 0.05). Sound-Glass-hybrid GIC-Junction and CMCR-Glass-hybrid GIC-Junction groups showed the lowest Aluminum levels and they showed significant differences with all test groups ((*p* < 0.05). Insignificant differences were found between Carious-Glass-hybrid GIC-Junction and Carious-Glass-hybrid GIC-Dentin groups and between CMCR-Bioactive-GIC-Junction and CMCR-Bioactive-GIC-Dentin groups (*p* > 0.05).

### Micro-raman microscopy

**Fig. 5:**
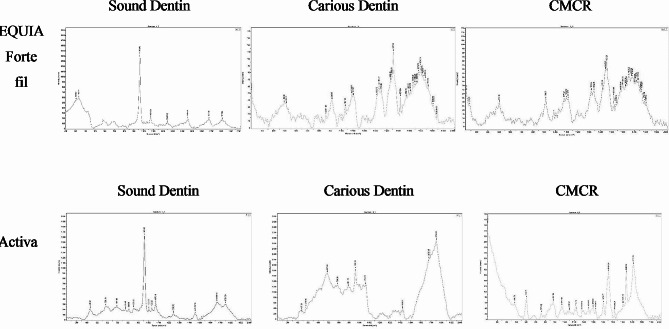
Micro-Raman spectroscopy of specimens showing the intensity of phosphate (v_1_ PO_4_^3−^) and carbonate (v_1_ CO_3_^2−^) bands at three different conditions

The major bands or parameters that can describe the mineral composition through micro-Raman spectroscopy corresponded to the phosphate (PO_4_^3−^) and carbonate (CO_3_^2−^) groups a shown in Fig. 5. Phosphate had two vibrations frequencies: major vibration frequency of v_1_ PO_4_^3−^ was near 960 cm^− 1^ and minor vibration frequencies of v_2_ and v_4_ PO_4_^3−^ vibrations were detected near 431 and 589 cm^− 1^, respectively. The vibration of v_1_ CO_3_^2−^ was detected near 1072 cm^− 1^. The high intensity of the v_1_ PO_4_^3−^ band is a positive sign and indicated high level of remineralization, while high intensity of the v_1_ CO_3_^2−^ is a negative sign and indicated high level of demineralization.

Regarding micro raman results: Bioactive-GIC specimens showed higher frequencies of v_1_ PO_4_^3−^ (higher than 960 cm^− 1^) and showed lower frequencies of v_1_ CO_3_^2−^(lesser than 1072 cm^− 1^) compared to Glass-hybrid GIC specimens. CMCR-Bioactive-GIC specimens showed the highest phosphate vibration frequency (971 cm^− 1^) and the lowest carbonate vibration frequency (1046 cm^− 1^). While Caries-Glass-hybrid GIC specimens showed the lowest phosphate vibration frequency (913 cm^− 1^) and the highest carbonate vibration frequency (1088 cm^− 1^). Comparing Sound-Glass-hybrid GIC specimens with Sound-Bioactive-GIC specimens: Sound-Bioactive-GIC specimen showed higher phosphate vibration frequency and lower carbonate vibration frequency (960,1070 cm^− 1^) than Sound-Glass-hybrid GIC specimens (940,1073 cm^− 1^). In addition, comparing Caries-Glass-hybrid GIC specimens with Sound-Bioactive-GIC specimens: Sound-Bioactive-GIC specimens showed higher phosphate vibration frequency and lower carbonate vibration frequency (960,1058 cm^− 1^) than Caries-Glass-hybrid GIC specimens (913,1088 cm^− 1^).

## Discussion

In dentistry, there has been an emerging trend towards the development of bioactive restorative materials and remineralizing resin-based materials are often mentioned as a possible way to increase the durability of bonded restorations. A new bioactive injectable resin modified self-adhesive glass ionomer restorative material has been recently introduced to dental field [11]. The manufacturer claimed that it could stimulate hydroxyapatite formation and natural remineralization at tooth-restoration interface through releasing and recharging Calcium, phosphate, and fluoride ions [12]. The manufacturer specified this material could be used for all direct anterior and posterior restoration, it can be utilized as an intermediate base [13]. This is attributed to surface precipitation of hydroxyapatite when exposed to water. Bioactive materials are able be biologically active or have a biological effect, and the ability to form a bond between the tissue and the material [14]. 

This bioactive restorative material was selected in this research to evaluate its effect on the microhardness, ions releasing property and its claimed bioactivity at the restoration-dentin interface in addition self-adhesion ability was also evaluated. The bioactive restorative material was compared with glass hybrid GIC that have no bioactive properties and consists of a highly viscous conventional GIC with ultrafine, reactive glass particles in addition to higher-molecular-weight poly acrylic acid molecules, which supposedly increase matrix cross-linking [15]. This offers a reasonable comparison to this bioactive restorative activity, thereby allowing practitioners to evaluate the restorative materials prognosis.

Minimally invasive caries removal is one of the most important applications of minimal intervention dentistry. Laser ablation, air abrasion, sono-abrasion or chemomechanical agent for the removal of infected dental tissue has been popular in minimal invasive caries removal techniques. These techniques are dependent on selective removal of caries-infected tissue, while leaving intact the caries-affected tissue. The ‘caries-infected’ dentin is the outer layer of dental caries that is completely deteriorated, has irreversible denaturation of the collagen fibres and cannot be remineralized. While ‘caries-affected’ dentin is the internal layer of dental caries that undergoes partial demineralization, deposition of crystals in tubules and the collagen fibrils are not destroyed. CMCR agents are used to dissolve the permanently damaged collagen fibers in infected dentin, facilitating their removal while avoiding the underlying healthy affected dentin [16]. . Papain enzyme based CMCR agent that was used in this study consists of papain enzyme, chloramine and toluidine blue. Papain is a natural product; patented, registered and approved by ANVISA in Brazil, it is a cysteine protease derived from the fruits and latex of green papaya (*Carica papaya*). Papain enzyme efficiently remove infected tissue due to the lack of plasmatic anti protease called Anti-trypsin. Antitrypsin role, is to inhibit protein digestion and presents only in sound uninfected tissues. Papain is thought to work by causing the breakdown of partly degraded molecules of collagen and aiding the disintegration and eradication of the mantle of the fibrin generated by the carious process without damaging the unimpaired collagen fibrils. As a result, the infected dentin becomes softer, making it possible to remove it without anesthetic and with non-cutting tools. This specific interaction has been explained by the absence of α-1-antitrypsin, a plasmatic protease inhibitor in infected dentin. Chloramines have bactericidal and disinfectant properties, and have been demonstrated to inBioactive-GICte gram positive and gram negative bacteria. Toluidine blue was found to be effective against *Streptococcus mutans*. It is a photosensitive pigment that fixes into the bacterial membrane [9, 17]. . Most of the trials (∼ 85%) utilized the NaOCl-based chemomechanical agent due to its popularity. Conversely, only a few clinical trials used the papain based chemomechanical caries removal agent [18, 19]. 

The Vickers microhardness test is one of the most widely used tests to measure the mechanical properties and structural integrity of materials or substrates. Such an easy, simple, and nondestructive approach as it requires a limited area of the specimen to be tested. The specimen surface is impressed with a pyramidial-diamond indenter at a certain load for a certain period [20]. After removing the load, the diagonal imprint is measured using an optical microscope to determine the size of the imprint. Microhardness analysis of residual dentin following caries removal has been useful to measure mineral loss and to detect whether demineralization or remineralization had occurred within the residual dentin. The decrease in micro-hardness value has been an indicative for mineral loss from the tooth structure, which is a sign for demineralization of the affected dentin [21, 22]. . Accordingly, the first null-hypothesis was rejected as all the variables used to compare Vickers micro-hardness values had a significant effect. The hardness of the residual dentin in (either sound dentin or carious dentin left or removed previously using papain based CMCR agent) exhibited minor changes among the different indentation points from the floor of the excavated surface. However, in all groups, dentin microhardness gradually decreased towards the dentin-restoration junction edge except for sound bioactive GIC group and CMCR-bioactive GIC group that showed increase in dentin micro-hardness. This is an indication that chemomechanical excavation method provided a high degree of collagen exposure, enhanced hybridization with the restorative material and maintain dentin irregular surface.

Similar outcomes were found in a study that had evaluated dentin microhardness after application of papain-based enzymatic CMCR agent, which resulted that it had preserved the dentin structure and microhardness [23]. In this study bioactive resin-modified glass ionomer showed higher dentin Vickers micro-hardness value than the glass hybrid glass ionmer material in most of the groups especially at the junction of the restoration and dentin that also showed significant differences. Increase Vickers microhardness of Bioactive-GIC may be due increase of resin monomer ratio which may had led to increase in wear resistance and hardness of the material [24]. The increase of microhardness values at the junction of dentin with Bioactive GIC could also be due presence of high percentage of silica that was proved after elemental analysis using EDX. The use EDX software ‘area selection’ tool is considered as an accurate method in detection of minor variations in mineral content among intimately close zones (e.g. superficial and sub- surface dentin or hybrid layers) [25]. The selection of detected elements in the current study was determined based on the marker ‘unique element’ of each material or substrate [26]. The aluminum is considered the marker of conventional GIC, while phosphorus is the unique element of tooth substrate [25]. Silica promotes remineralization of exposed dentinal tubules by obstruction of exposed dentinal tubule, as silica helps in increasing crystallization of the material [27, 28]. Bio-silicate (P_2_O_5_–Na_2_O–CaO–SiO_2_), A fully crystallized bioactive glass-ceramic has been proposed to deposit hydroxyl carbonate apatite in open dentinal tubules, studies have reported complex sequence of reactions that takes place on immersion in a simulated body fluid and a fast ion exchange starts between the alkaline ions from the glass surface and the hydrogen ions from the solution that leads to formation of free − OH groups (silanols) that is followed by polycondensation developing a silica gel layer. This layer stimulates the adsorption of calcium and phosphorous ions from the solution to induce hydroxyapatite deposition, this bioactive film was found to be resistant to abrasion and erosion. In addition, silanol was proved to be the active site of interaction between the silica surface and the phosphate-charged groups of phospholipids [28, 29]. 

The interface between the restoration and the tooth structure micromorphological patterns are considered one of the most important factors in restoration durability. The interdiffusion zone acts as a protective layer for the tooth, preventing microorganisms and their toxins from entering the dentin/pulp tissues. This hybrid layer is formed through impregnation of resin ingredients into the exposed collagen network following demineralization of dentin, producing micromechanical interlocking, penetration of resin into the dentinal tubules forms resin tags. One of the methods used to evaluate the hybrid layer is the environmental scanning electron microscope. Elemental analysis is conservative, nondestructive and simple to study mineral composition within narrow areas of tissues, it is a theoretical method based on mathematical methodology. EDX mapping was used to identify the elemental composition of the material interface [30]. In this study the mineral content (calcium, phosphorus, silica, aluminum) of normal dentin has been estimated by EDX were in the same range as in previous studies. The mineral content of dentin was unaffected by papain based-enzymatic CMCR agent as demonstrated in previous studies [31]. Hence, the null-hypothesis was rejected; there was no significant difference in the chemical composition of dentin in the three study groups. While the elemental analysis at the interface between restorative materials and dentin showed differences between the study groups. Calcium and Phosphorous analysis at the junction estimated that mainly the glass hybrid glass ionomer cement showed higher mean value than the bioactive resin modified glass ionomer, but at the same time they were insignificantly different. The main reason for decrease of calcium and phosphate weight% may be due the higher base of the UDMA polymeric resin matrix of the bioactive resin modified glass ionomer while no resin structure was found in the other restorative material. Silica and Aluminum mean value at the junction between the restorative material and dentin were significantly higher with bioactive resin modified glass ionomer than the glass hybrid restorative material, therefore the null-hypothesis was rejected in this point. Increase the silica released from the bioactive resin modified glass ionomer at the interface confirms the higher value of microhardness of bioactive GIC than the glass-hybrid GIC, due to silica was proved to increase the bioactivity and abrasion resistance as mentioned before.

In order to confirm VMH test and EDX results one specimen from each study group was subjected to micro-Raman spectroscopy. Raman spectroscopy is a quantitative, analytical and nondestructive approach that can detect the molecular level and composition by irradiating the specimen with a visible laser source. Micro-Raman spectroscopy revealed that Bioactive GIC specimens showed higher frequencies of v_1_ PO_4_^3−^ and lower frequencies of v_1_ CO_3_^2−^, which is a is a positive sign and indicated high level of remineralization. Micro-Raman spectroscopy also supported EDX findings, as the highest v_1_ PO_4_^3−^ reveals superior in remineralization and penetration at the restoration-dentin interface [10]. 

In light of the current study the null hypotheses were rejected. Accordingly, future studies are necessary to assess the bioactive properties of the ion releasing claimed bioactive GIC with the application of bonding agents, and to confirm that using a bonding will not be as barrier for bioactivity and remineralization process.

## Conclusions

It was concluded that ion-releasing bioactive resin-based restorative material seems to be promising restorative materials of caries-affected dentin. Enzymatic caries excavation with papain-based CMCR agent has no adverse effect on dentin substrate.

### Electronic supplementary material

Below is the link to the electronic supplementary material.

Supplementary Material 1

Supplementary Material 2

## Data Availability

The datasets used and/or analysed during the current study are available from the corresponding author on reasonable request.
